# Addressing Key Limitations of Diastolic Function Assessment in Mouse Echocardiography by Enabling Robust Retrospective Analysis From a Standard Imaging View

**DOI:** 10.1111/apha.70250

**Published:** 2026-05-14

**Authors:** Michael Marterstock, Antje Schauer, Alina Maslakova, Annett Opitz, Peter Dieterich, Stephan Speier, Susanne Kämmerer, Peter Mirtschink, Irakli Kopaliani, Andreas Deussen

**Affiliations:** ^1^ Institute of Physiology Faculty of Medicine, TUD Dresden University of Technology Dresden Germany; ^2^ Laboratory of Experimental and Molecular Cardiology, Department of Internal Medicine and Cardiology Heart Centre Dresden|Faculty of Medicine, TUD Dresden University of Technology Dresden Germany; ^3^ Institute of Pharmacology and Toxicology Faculty of Medicine, TUD Dresden University of Technology Dresden Germany; ^4^ Institute for Clinical Chemistry and Laboratory Medicine Faculty of Medicine, TUD Dresden University of Technology Dresden Germany

**Keywords:** diastolic function, echocardiography, HFpEF, HFrEF, mouse

## Abstract

**Aim:**

Echocardiographic assessment of diastolic function in mice remains challenging because parameters translated from clinical practice are constrained by murine physiology. In particular, parameters derived from early (*E*) and late (*A*) transmitral filling velocities are frequently compromised by wave fusion at higher heart rates. Here, we quantified these limitations and evaluated an alternative method for measuring isovolumetric relaxation time (IVRT), a marker reflecting ventricular relaxation and a key element of established algorithms specific for murine diastolic function assessment.

**Methods:**

In a non‐A4C‐view‐dependent echocardiographic measurement (NAEM) approach, speckle‐tracking analysis of standard brightness‐mode (B‐mode) cine loops from the parasternal long‐axis view (PSLAX) enabled *E*/*A* ratio and IVRT assessment. Mice with unimpaired heart function, heart failure with preserved ejection fraction (HFpEF), or heart failure with reduced ejection fraction (HFrEF) (*n* = 8 each) underwent standard Doppler‐based and NAEM‐based measurement of *E*/*A* ratio and IVRT.

**Results:**

In mice with unimpaired heart function and HFpEF, heart rate‐dependent *E* and *A* wave fusion occurred at an estimated threshold of approximately 460 bpm, whereas advanced HFrEF led to frequent fusion independent of heart rate. NAEM‐derived IVRT showed strong agreement with the Doppler reference (*r* ^2^ = 0.9339, *p* < 0.0001), minimal bias (−1.4 ms), and similar inter‐observer variability. In contrast, NAEM‐derived *E*/*A* ratio did not show adequate performance.

**Conclusion:**

These findings further support the shift toward specific algorithms for diastolic function assessment in murine echocardiography. The validated NAEM approach supports application of such protocols without dedicated Doppler recordings by using PSLAX B‐mode cine loops, thereby lowering technical barriers for future studies and enabling robust retrospective analysis of existing datasets.

## Practitioner Points

- Novel measurement approach addresses previous limitations in murine echocardiography.
- Complementing an established comprehensive algorithm for diastolic function assessment.
- Enabling retrospective analysis of pre‐existing datasets using a common echo view.

## 1 Introduction

Mice serve as primary disease models in cardiovascular basic research [1, 2, 3]. In that context, echocardiography has emerged as an indispensable tool for repetitive, in vivo, and noninvasive assessment of cardiac function [1, 2, 3, 4, 5]. However, while echocardiographic protocols for systolic function assessment are well standardized and widely applied, the assessment of diastolic function has gained significant attention only recently due to clinical insights in human heart failure with preserved ejection fraction (HFpEF) but impaired diastolic function [2, 6]. Accordingly, robust assessment of diastolic performance in murine animal models has become an increasingly important objective in cardiovascular research.

Here, we address three principal challenges that currently limit the robustness and broader applicability of diastolic function assessment in mouse echocardiography. First, diastolic phenotyping relies on technically demanding imaging approaches that require advanced operator expertise. Second, intrinsic physiological constraints of the murine heart, most notably its high heart rate, frequently result in fusion of early (*E*) and late (*A*) transmitral filling waves, thereby limiting the interpretability of classical Doppler‐based parameters. Third, the requirement for dedicated imaging views and Doppler recordings precludes retrospective diastolic analyses of a large number of existing echocardiographic datasets primarily acquired for systolic assessment.

The first major limitation arises from the technical requirements of diastolic echocardiographic imaging in mice. While most systolic function parameters can be measured in brightness mode (B‐mode) images of the parasternal long axis (PSLAX), for diastolic function assessment it is crucial to image the flow velocity over the mitral valve (MV). The gold standard approach in this is to use Pulsed wave Doppler (PW Doppler) cine loops of the apical four‐chamber view (A4C), with the gate placed inside the left ventricle, in close proximity to the MV [2, 3, 4, 7]. Unfortunately, while the B‐Mode PSLAX recordings require moderate experience, imaging the mitral flow profile from the A4C view is considered technically more demanding, which excludes operators new to murine echocardiography from diastolic function studies until a lengthy training period is met [4, 5].

This challenge is partially due to the carefully angled positioning of the transducer but more significantly because of the intrinsic physiological properties of the murine heart, which give rise to the second limiting factor, namely the high heart rate.

In comparison to larger mammals like humans, the murine heart is very small in size (5–8 mm length in adult mice [3]) and has a significantly higher heart rate (> 600 beats per minute [bpm] conscious, > 400 bpm anesthetized [3, 4, 8, 9, 10]), leading to short diastolic filling periods. This heavily affects the assessment of diastolic function parameters such as the *E*/*A* ratio, a classical parameter for diastolic function translated from clinical practice [11, 12]. In murine disease models and especially at higher heart rates, the *E* and *A* wave fuse partially or totally, making a meaningful measurement of the *E*/*A* ratio impossible [2, 4, 7, 8, 9, 13]. Various sources describe estimated threshold values for this phenomenon ranging widely from 400 to 600 bpm, but to date, there is no differentiation between disease models and no satisfactory quantitative presentation of the underlying data [3, 4, 7, 9].

In addition, *E*/*A* fusion may also compromise the interpretation of other established diastolic parameters. The *E*/*E*′ ratio, the early mitral peak filling velocity *E* divided by peak mitral annular velocity *E*′ determined by tissue Doppler, is another widely established echocardiographic marker of diastolic dysfunction in human care as in basic research [2, 4, 7, 11, 12]. But again, it is unclear whether the murine *E*/*E*′ measurement is meaningful in cases of partial or total *E*/*A* fusion, especially since the early and late peak mitral annular velocities (*E*′ and *A*′) do not strictly merge synchronously with *E* and *A*. This circumstance has not been sufficiently examined in the available literature to date.

Limitations of clinical parameters in murine echocardiography have been addressed in one of the current benchmark protocols for diastolic function assessment in mice [7]. This protocol provides a comprehensive algorithm specific for murine echocardiography and independent from the *E*/*A* or *E*/*E*′ ratio integrating left atrial area (LAA), isovolumetric relaxation time (IVRT) and (reverse peak) longitudinal strain rate ((r)LSR). In that, increased LAA proved to be a sensitive indicator of elevated left atrial pressure in several murine models of chronic diastolic dysfunction. Furthermore, IVRT and (r)LSR allow for characterization of the diastolic phenotype. IVRT, corresponding to the time interval within the cardiac cycle just before MV opening, but after aortic valve closure, may indicate a diastolic dysfunction due to impaired relaxation of the left ventricle when prolonged, but point to a restrictive filling pattern when shortened [7]. As (r)LSR serves as an indicator for the peak early filling rate of the left ventricle, pathological alterations are reciprocal to the changes of IVRT. Although these three parameters allow for a comprehensive insight in murine diastolic performance [7], two of the three limitations noted above, namely the investigator's experience and the potential for retrospective data analysis, remain. Conducting retrospective diastolic function analyses of previously conducted studies on systolic function is often not feasible, since data primarily consist of B‐Mode PSLAX cine loops and frequently do not include the prior described and strictly required A4C view recordings.

**FIGURE 1:**
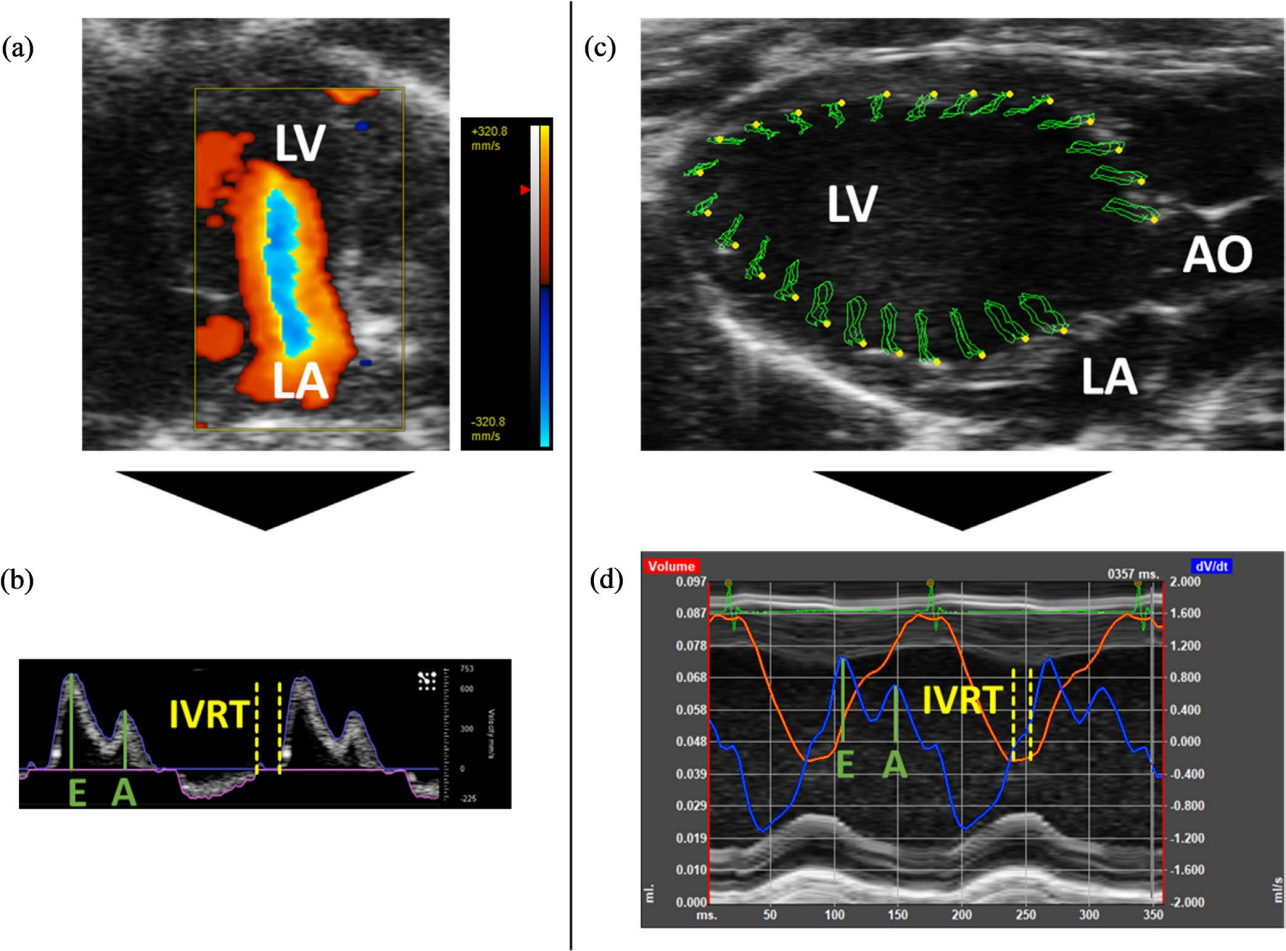
Illustration of the gold standard approach for *E*/*A* ratio and IVRT measurement versus the NAEM approach. (a) Representative image of an A4C view. Left ventricle (LV) and left atrium (LA) are labeled for orientation. Mitral inflow is visualized by the Color Doppler application. (b) The corresponding PW Doppler signal, peak passive (*E*), and active (*A*) flow velocities are marked in green, IVRT is represented by the interval between the dotted vertical lines marked in yellow. (c) Representative image of a PSLAX view with clear visualization of the apex cordis as well as the aortic valve. Left ventricle (LV), left atrium (LA), and aortic outflow tract (AO) are labeled for orientation. Myocardial movement and strain are tracked via speckle‐tracking, here shown by the orbit lines (green) of 25 ROIs over two cardiac cycles. (d) Speckle‐tracking derived LVV(t) curve (orange) and *dLVV*(*t*)/*dt* curve (blue). Labeling of peak passive (*E*) and active (*A*) flows and IVRT as given in (b).

Whereas LAA [2, 4, 14] and rLSR [2, 7, 15] can be quantified from B‐Mode PSLAX cine loops, IVRT measurements again require MV flow profile recordings using A4C view PW Doppler cine loops. Hence, determination of IVRT interval indicated by time sections where flow velocity is equal to zero (Figure 1a,b) [2, 3, 4].

Therefore, the aims of the present communication are threefold: First, to quantify a threshold heart rate above which *E* and *A* wave fusion may occur to determine a reliable range for assessing diastolic function in the mouse heart in vivo. Second, by building on the Schnelle algorithm [7] to evaluate a Non‐A4C‐view‐dependent Echocardiographic Measurement (NAEM) based on speckle tracking analysis of B‐Mode PSLAX cine loops. Finally, to test if the NAEM approach offers a reliable option in *E*/*A* ratio assessment or whether this approach shares the same limitations in regard to *E* and *A* wave fusion as the standard approach.

## 2 Results

To investigate *E* and *A* wave fusion and to validate the NAEM approach for different states of impaired diastolic function, three different models of diastolic (dys)function were selected (Table 1). Unimpaired diastolic function was observed in mice with normal heart function. Chronic moderate diastolic dysfunction was observed in a murine model of HFpEF, and acute severe diastolic dysfunction was observed in a model of heart failure with reduced ejection fraction (HFrEF). These were examined in groups of eight mice each following the indicated echocardiography protocol (as described in Section 4.3).

**TABLE 1:** Phenotyping measurements of mouse models with different states of cardiac dysfunction.

Unimpaired heart function	No treatment (*n* = 8)
BW (g)	24.2 ± 2.3
HR (bpm)	426.50 ± 35.20
EF (%)	57.73 ± 7.04
GLS (%)	−20.79 ± 2.78
LV Mass Cor (mg)	79.46 ± 14.97
RWT	0.36 ± 0.08
LAA (mm^2^)	3.00 ± 0.43
IVRT (ms)	11.21 ± 0.89
(r)LSR	6.57 ± 1.64
E/E'ratio	# 20.85 ± 0.83
E/A ratio	# 1.61 ± 0.10

HFpEF	Normal diet (*n* = 6)	HFD + L‐NAME (*n* = 8)
BW (g)	28.7 ± 1.8	29.9 ± 1.5
HR (bpm)	466.50 ± 26.16	434.50 ± 35.14
EF (%)	48.91 ± 4.96	48.27 ± 7.45
GLS (%)	−16.56 ± 2.05	−14.80 ± 3.69
LV Mass Cor (mg)	106.20 ± 23.46	101.10 ± 14.02
RWT	0.35 ± 0.13	0.33 ± 0.06
LAA (mm^2^)	3.53 ± 0.33	3.90 ± 0.22*
IVRT (ms)	12.46 ± 0.57	16.49 ± 4.16*
(r)LSR	5.26 ± 0.97	(*p* = 0.07) 4.00 ± 1.19
*E*/*E*′ ratio	23.13 ± 5.37###	31.77 ± 6.48*
*E*/*A* ratio	1.59 ± 0.22###	1.36 ± 0.26##

HFrEF	PBS—injected (*n* = 8)	LPS—injected (*n* = 8)
BW (g)	23.3 ± 3.1	23.4 ± 2.6
HR (bpm)	425.10 ± 28.82	(*p* = 0.08) 457.50 ± 34.60
EF (%)	53.81 ± 4.51	25.55 ± 4.25****
GLS (%)	−15.36 ± 2.26	−7.49 ± 2.78****
LV Mass Cor (mg)	78.69 ± 12.63	84.68 ± 11.42
RWT	0.33 ± 0.06	0.40 ± 0.05*
LAA (mm^2^)	3.380 ± 0.46	3.27 ± 0.41
IVRT (ms)	14.34 ± 1.53	30.21 ± 6.36****
(r)LSR	4.594 ± 0.64	2.73 ± 0.36****
*E*/*E*′ ratio	# 25.47 ± 3.70	26.89 ± 5.02
*E*/*A* ratio	1.53 ± 0.18	No usable data####

*Note:* Echocardiographic analysis was done by one of the three observers for all data sets. Assessed parameters of systolic function: body weight (BW) in grams, heart rate (HR) in beats per minute, ejection fraction (EF) in %, and global longitudinal strain (GLS) in %; morphology: left ventricular mass (corrected [4]) (LV Mass (Cor)) in milligrams and relative wall thickness (RWT) (= 2 × enddiastolic left ventricular posterior wall thickness/enddiastolic left ventricular inner diameter); diastolic function: left atrial area (LAA) in square millimeter, isovolumetric relaxation time (IVRT) in milliseconds, (reverse peak) longitudinal strain rate (r)LSR, *E*/*E*′ ratio and *E*/*A* ratio. Due to *E* and *A* wave fusion mice were excluded from the E/A ratio analysis as described in Section 2.3.; #*n* = 7, ##*n* = 6, ###*n* = 4, ####*n* = 0. EF, GLS, LAA, and (r)LSR were determined from B‐Mode PSLAX cine loops. LV Mass (Cor) and RWT were determined from M‐Mode PSSAX cine loops. HR, IVRT, *E*/*E*′ and *E*/*A* ratio were determined from PW Doppler A4C cine loops. Values are presented as mean ± standard deviation, unpaired *t*‐test was used to detect significance. The shown data originates from different experimental series with varying ages and mouse substrains, thus parameters may vary between control groups and treatment groups should be only compared to their respective controls.

*p < 0.05 ****p < 0.0001 versus respective controls.

Heart rate‐dependent *E* and *A* wave fusion was characterized in the PW Doppler recordings of the A4C view by relating the temporal interval between *E* and *A* wave peak to heart rate.

IVRT and *E*/*A* ratio were assessed according to published standards, using PW Doppler recordings of the A4C view (referred to as Gold Standard‐IVRT and Gold Standard‐*E*/*A*) [2, 3, 4, 7], as well as with the novel NAEM approach (referred to as NAEMI‐IVRT and NAEM‐*E*/*A*) to be evaluated here (Figure 1c,d). The parameters assessed by the novel NAEM approach were then compared to those measured by the gold standard approach (PW Doppler of A4C view), for each experimental group separately as well as in a combined analysis.

### 2.1 Relation of *E* and *A* Wave Fusion to Heart Rate

**FIGURE 2:**
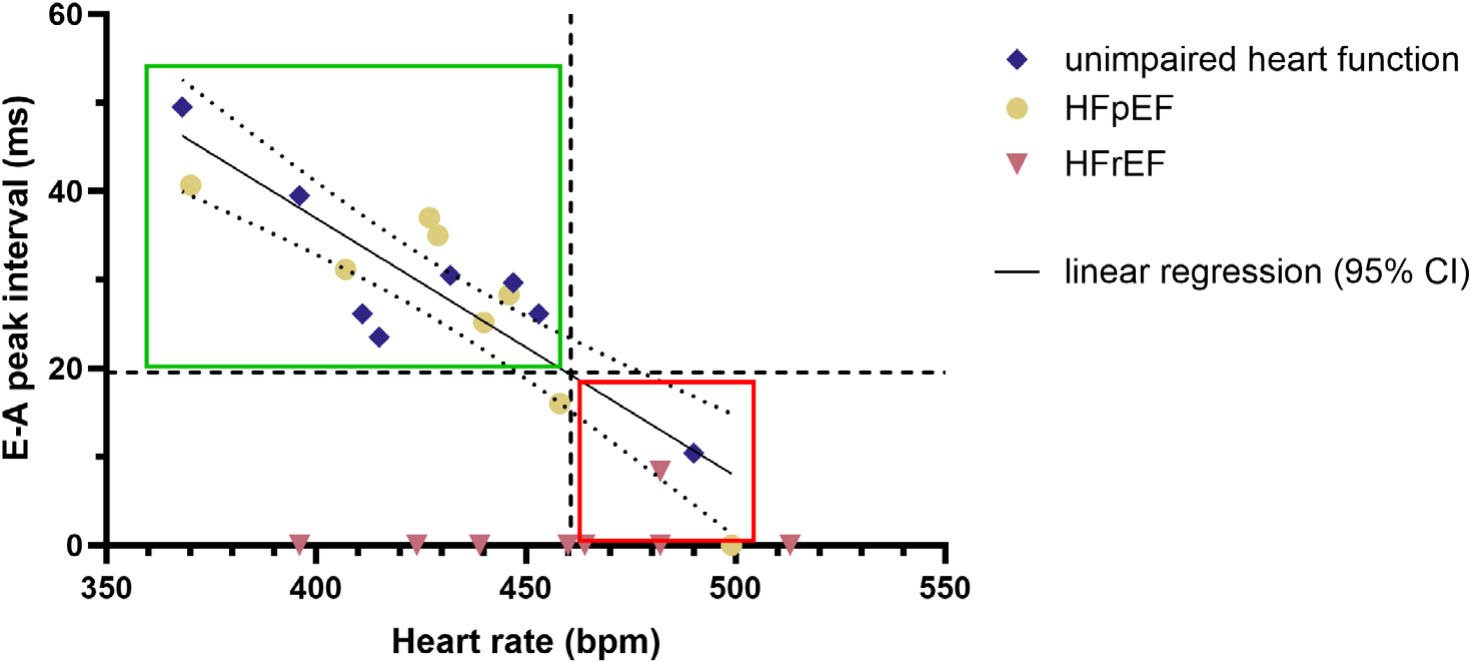
Relation of *E* and A wave fusion to heart rate. Depicted as single values of *E*–*A* peak interval (milliseconds) to heart rate with 8 data points per experimental group. Linear regression (solid line) + 95% confidence intervals (dotted lines) for combined data from unimpaired heart function and HFpEF group only. Shift from separated *E* and *A* wave to partially fused waves is depicted as horizontal broken line. Estimated threshold heart rate for fusion of *E* and *A* wave is depicted as vertical broken line (460.73 bpm as mean of 446.16 and 471.88 bpm). Estimated range of robust *E*/*A* ratio measurement is indicated as green box, estimated range of unfeasible *E*/*A* ratio measurement is indicted as red box. In HFrEF, the correct *E*–*A* peak interval could not be assessed in seven out of eight experiments due to peak fusion independent of heart rate (triangles on abscissa). *Note:* This analysis is intended to demonstrate the feasibility of this approach. The estimated heart rate threshold may depend on strain, model and condition and it is not intended to be used as a universally applicable cut‐off value.

In mice with unimpaired cardiac function and HFpEF a progressive shortening of the *E*–*A* peak interval correlated significantly with increasing heart rate, leading ultimately to partial (for one animal each or 12.5% in both groups) or total fusion (for one animal or 12.5% in the HFpEF group) (Figure 2, Table 2). In contrast, in HFrEF mice, the *E* and *A* waves were typically fused with one partial fusion (12.5% of examined animals) and seven total fusions (87.5% of examined animals) even at moderate heart rates. Fusion occurred without a clear relation and precluded meaningful further regression analysis (Figure 2, Table 2). Noteworthy, classification of *E* and *A* wave fusion in this analysis was based on the assessment of one experienced observer and therefore differs from the observer consensus used for fusion classification as described in Section 2.3. Values for the groups of unimpaired heart function and HFpEF were further used for regression analysis. A simple linear regression proved appropriate (unimpaired heart function *r* ^2^ = 0.7650, HFpEF *r* ^2^ = 0.8009). In an analysis of covariance (ANCOVA) no significant differences in slope (*F* = 0.2932, *p* = 0.5981) or elevation (*F* = 0.0201, *p* = 0.8895) were found between both curves. Data of both groups were taken together for a combined linear regression analysis (*r* ^2^ = 0.7829) to identify a threshold heart rate for *E* and *A* wave fusion (Fig. 2, Table 2). By solving the generated equation of this curve (*y* = −0.2916*x* + 153.6; *y*: *E*–*A* peak interval, *x*: heart rate) for the lowest measured peak interval without fusion and the highest peak interval with partial fusion, a range for this threshold could be estimated between 446.16 and 471.88 bpm (Fig. 2).

**TABLE 2:** Results of Pearson's correlation for *E*–*A* peak interval to heart rate comparison.

	Unimpaired heart function (*n* = 8)	HFpEF (*n* = 8)	HFrEF (*n* = 8)	Unimpaired heart function and HFpEF combined (*n* = 16)
Pearson's correlation				
*p*	0.0045	0.0027	0.5216	< 0.0001
*r* ^2^	0.7650	0.8009	0.0716	0.7829
*r* Confidence interval (95%)	−0.9771 to −0.4430	−0.9810 to −0.5152	−0.5286 to 0.8180	−0.9596 to −0.6932
Simple linear regression analysis				
Slope	−0.2671	−0.3148	/	−0.2916
Slope confidence interval (95%)	−0.4150 to −0.1192	−0.4716 to −0.1580	/	−0.3796 to −0.2036
*y*‐intercept	143.4	163.5	/	153.6
*x*‐intercept confidence interval (95%)	80.06 to 206.6	95.11 to 231.8	/	115.6 to 191.6

*Note:* Data of one observer with 8 animals per experimental group.

### 2.2 Comparison of IVRT Measurement

**FIGURE 3:**
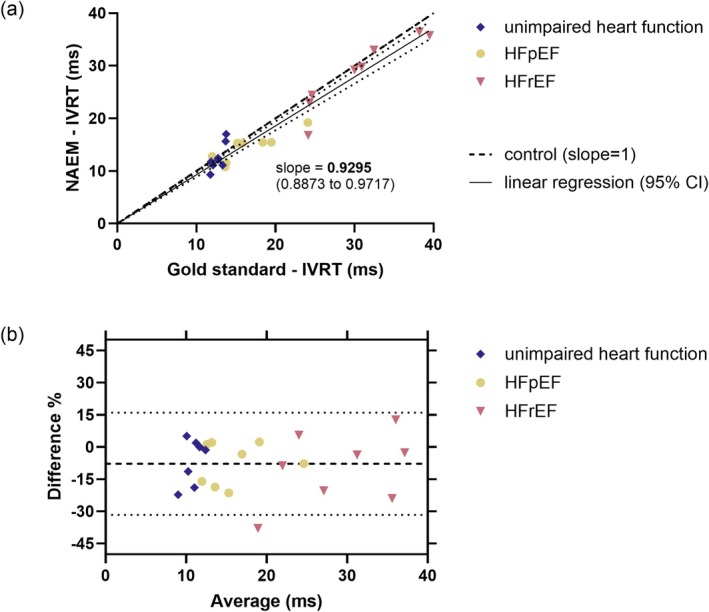
Comparison of gold standard approach versus NAEM approach for IVRT measurement. Depicted for the combined analysis of averaged values of three observers (for individual data see Figure S1) with 8 data points for data of eight animals per experimental group. All observers used identical imaging data for their retrospective analysis. (a) Individual data points; linear regression forced through point of origin (solid line) + 95% confidence intervals (dotted lines); line of identity (*y* = *x*, slope = 1) (broken line). (b) Bland–Altman plot showing the reproducibility of NAEM approach versus gold standard IVRT measurement as difference of the NAEM approach (%) against the average of both measurements; the average bias of the combined analysis of the NAEM approach is represented by the broken line; 95% limits of agreements shown by dotted lines.

In all three experimental groups, as well as in a combined analysis, IVRT values obtained using the NAEM approach correlated significantly with their counterparts of the gold standard measurement (Figure 3a, Table 3) (for individual/not averaged data points see Figure S1 and Table S1). The bias of the NAEM approach was −0.2 ms (−2.9%) for animals with unimpaired heart function, −2.1 ms (−12.5%) in HFpEF animals, −2.0 ms (−7.7%) in HFrEF animals and on average −1.4 ms (−7.7%) in a combined analysis (Figure 3b, Table 3). The coefficient of variation (CV) of interobserver variability was calculated to be 15.1% for the gold standard measurement and 17.0% for the NAEM approach. The CV for variability between the two approaches was calculated to be 8.0%. Notably, only for the analysis of one animal was one of the three observers not able to measure IVRT using the NAEM approach.

**TABLE 3:** Results of Pearson's correlation and Bland–Altman analysis for IVRT measurement comparison.

	Unimpaired heart function (*n* = 8)	HFpEF (*n* = 8)	HFrEF (*n* = 8)	Combined (*n* = 24)
Pearson's correlation				
*p*	0.0151	0.0039	0.0008	< 0.0001
*r* ^2^	0.6538	0.7743	0.8640	0.9339
*x* confidence interval (95%)	0.2416 to 0.9640	0.4614 to 0.9781	0.6518 to 0.9874	0.9226 to 0.9856
Bland–Altman analysis: NAEM approach vs. average				
Bias (%)	−2.9	−12.5	−7.7	−7.7
SD of bias (%)	14.7	11.9	11.9	13.0
Bias (ms)	−0.18	−2.066	−1.97	−1.40
SD of bias (ms)	1.94	2.02	2.51	2.26

*Note:* Mean data of three observers with eight animals per experimental group.

### 2.3 Comparison of the *E*/*A* Ratio Measurement

**FIGURE 4:**
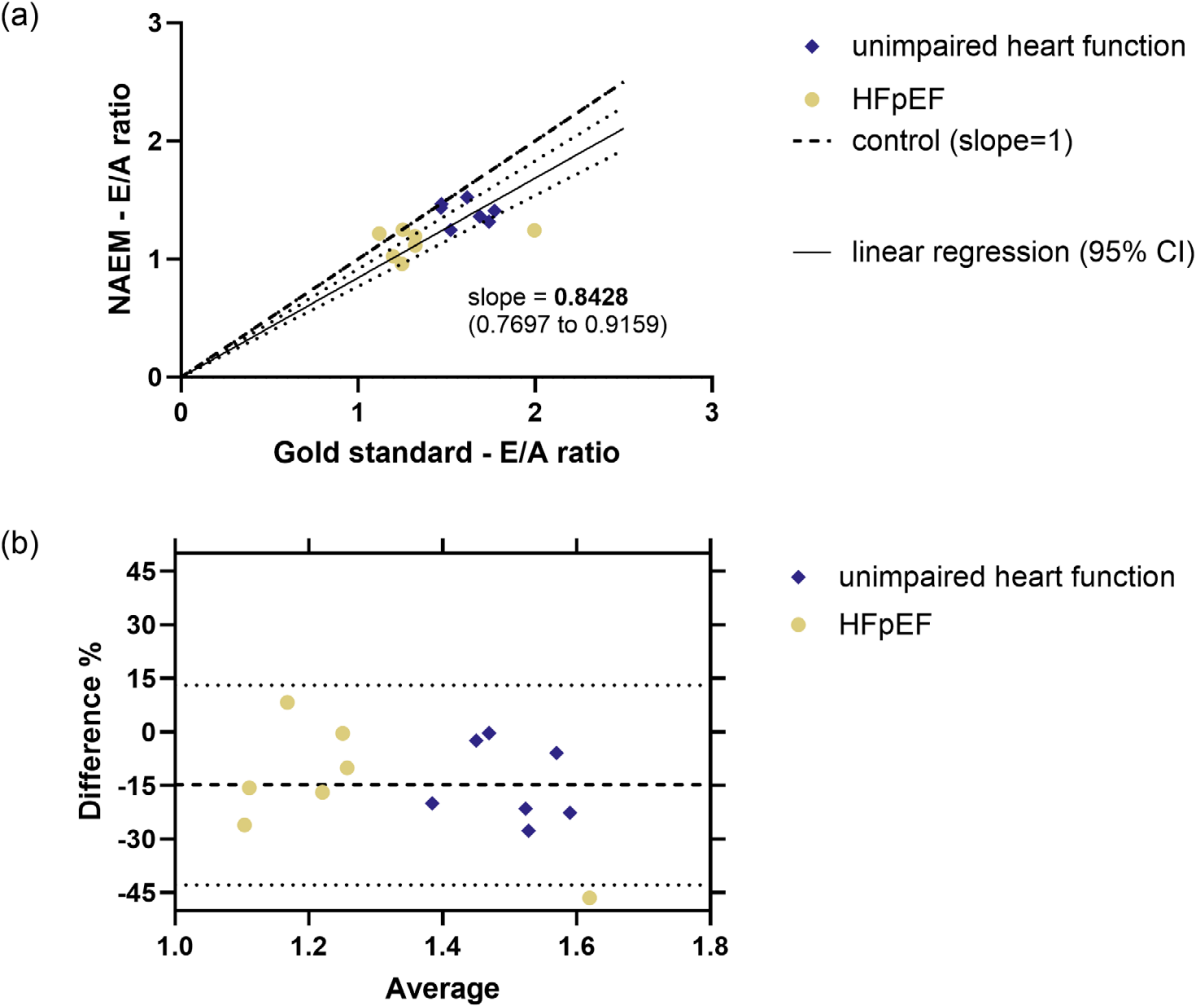
Comparison of gold standard approach vs. NAEM approach for *E*/*A* ratio measurement. Depicted for the combined analysis of averaged values of three observers with 7 data points for retrospective data analysis of seven animals per experimental group (data was partially excluded as described in Section 2.3). All observers used identical imaging data for their retrospective analysis. (a) Individual data points; linear regression forced through point of origin (solid line) + 95% confidence intervals (dotted lines); line of identity (*y* = *x*, slope = 1) (broken line). (b) Bland–Altman plot showing the reproducibility of NAEM approach versus gold standard *E*/*A* ratio measurement as difference of the NAEM approach (%) to the average of both measurements; the average bias of the combined analysis of the NAEM approach is represented by the broken line; 95% limits of agreements shown by dotted lines.

Due to the occurrence of *E* and *A* wave fusion, *E*/*A* ratio measurement was not feasible for all recordings. Accordingly, mice were excluded from the analysis if at least two of the three observers identified partial or total fusion of the *E* and *A* wave. In Gold Standard‐*E*/*A* ratio this was true for one mouse (12.5% of examined animals) in the experimental group of HFpEF and six mice (75% of examined animals) in the HFrEF group. In NAEM‐*E*/*A* ratio this was true for one mouse (12.5% of examined animals) in the experimental group with unimpaired heart function, as well as for two mice (25% of examined animals) of the HFrEF group. Because of this, the number of data pairs for both approaches was limited to seven for the group with unimpaired heart function as well as for the HFpEF group and to one data pair in the HFrEF group. Thus, a meaningful further analysis of the HFrEF group was not possible. With respect to the other two experimental groups, the correlation analysis of the datasets showed no significant correlation between *E*/*A* ratio values of the NAEM approach compared to the gold standard measurement for mice with unimpaired heart function, HFpEF mice, as well as for a combined analysis (Figure 4a, Table 4). In general, the *E*/*A* ratio values of the NAEM approach covered a narrower data range than the related gold standard measurements, which might indicate a lower sensitivity for detection of differences. The bias of the NAEM approach was −0.2167 (−14.3%) for animals with unimpaired heart function, −0.2078 (−15.3%) in HFpEF animals, and −0.2123 (−14.8%) in the combined analysis of both groups (Figure 4b, Table 4). The CV of inter‐observer variability was calculated to be 2.6% for the gold standard measurement and 15.9% for the NAEM approach. The CV for variability between the two approaches was calculated to be 11.3%.

**TABLE 4:** Results of Pearson's correlation and Bland–Altman analysis for E/A ratio measurement comparison.

	Unimpaired heart function (*n* = 7)	HFpEF (*n* = 7)	HFrEF (*n* = 0)	Combined (*n* = 14)
Pearson's correlation				
*p*	0.7194	0.4161	/	0.0505
*r* ^2^	0.0281	0.1358	/	0.2825
*x* confidence interval (95%)	−0.8175 to 0.6700	−0.5323 to 0.8779	/	0.0013 to 0.8285
Bland–Altman analysis: NAEM approach vs. average				
Bias (%)	−14.3	−15.3	/	−14.8
SD of bias (%)	11.1	17.8	/	14.3
Bias (absolute)	−0.2167	−0.2078	/	−0.2123
SD of bias (absolute)	0.1695	0.2724	/	0.2180

*Note:* Mean data of three observers with seven animals for unimpaired heart function and HFpEF, respectively, and no data points for HFrEF. Data were partially excluded as described in Section 2.3.

## 3 Discussion

In this study, three central limitations of diastolic function assessment in murine echocardiography were addressed, namely the physiological constraints imposed by heart rate‐dependent filling dynamics, the high technical demands of established imaging approaches, and the lack of retrospective analysis options for existing datasets. Among these limitations, fusion of *E* and *A* waves represents a known but largely under‐quantified factor, as it directly compromises the interpretability of widely used transmitral Doppler parameters such as *E*/*A* ratio. The present analysis provides a quantitative assessment of the relation of heart rate to *E* and *A* wave fusion in murine echocardiography. The estimated heart rate of approximately 460 bpm at which fusion occurred in mice with unimpaired cardiac function and HFpEF is consistent with previously reported values (heart rate of > 450 bpm), which have largely remained descriptive and lacked systematic quantification [4, 7]. In contrast, the findings of the HFrEF group regarding *E* and *A* wave fusion indicate that disease‐related alterations of diastolic filling may outweigh chronotropic effects in advanced cardiac dysfunction, limiting the interpretability of *E* and *A* wave‐based parameters even further and, potentially, for entire categories of disease models.

Importantly, this study represents an exploration toward a systematic and quantitative description of the phenomenon of *E* and *A* wave fusion in mice since limited group sizes as well as model and strain diversity [16] preclude broad generalization. However, it would be possible, by following this approach, to validate methods in other experimental studies.

The findings from three cardiac disease models highlighted clearly the limitations of the *E*/*A* ratio as a parameter of diastolic dysfunction, underlining the necessity to critically examine whether the *E*/*A* ratio measurement is feasible in animal models before conducting murine echocardiography studies or resorting to alternative parameters. Consequently, the need for diastolic assessment strategies that are independent of transmitral *E* and *A* wave morphology, such as the protocol proposed by Schnelle et al. [7], is reinforced.

To bypass this difficulty and to address the other two outlined limitations, this study introduced and evaluated a novel method of IVRT measurement, completing the algorithm for diastolic function assessment otherwise dependent on parameters of the transmitral flow profile [7]. The novel speckle tracking‐based NAEM approach for IVRT measurement demonstrated a strong correlation with the established gold standard approach based on PW Doppler A4C cine loops, exhibiting excellent performance in a point‐to‐point analysis as evidenced by the Bland–Altman plot (Figure 3b, Table 3). Moreover, with a CV of 8.0% for the variability between the two measurement approaches, NAEM resembles the gold standard closely without major disadvantages in inter‐observer variability (CV = 17.0% for NAEM‐IVRT and 15.1% for the gold standard). Due to the observed stable biases of IVRT measurement throughout groups with varying extents and dynamics in diastolic dysfunction, this novel approach is applicable for measurements in a wide range of IVRT, and thus, to a considerable fraction of common disease models.

In contrast to IVRT, the NAEM approach for *E*/*A* ratio measurement showed no significant correlation with the gold standard and challenges in reproducibility, indicating insufficient methodological equivalence.

Consequently, as in the Schnelle protocol [7] LAA and rLSR are already accessible from these recordings, the validated IVRT measurement now allows for full application of the suggested diastolic function assessment algorithm using exclusively B‐Mode PSLAX cine loops. This not only lowers technical barriers for future studies but also paves the way for re‐interpreting data from well‐established mouse models. By applying NAEM‐IVRT to archival datasets, researchers may uncover previously inaccessible diastolic function alteration without repeating experiments—potentially revealing new pathophysiologic mechanisms of diastolic dysfunction hidden in plain sight. With respect to the principle of the 3R (replace, reduce, refine) [17] this may also contribute to a reduction in repeating (now) unnecessary animal experiments.

Yet, the application of this approach is contingent upon the fulfillment of several conditions and is subject to certain limitations. In order to employ speckle‐tracking analysis, it is imperative that the B‐mode cine loops are of high quality (as to be seen in Figure 1c). Specifically, the recordings must encompass the entirety of the left ventricle, from the aortic valve to the apical epicardium and the left atrium with the endo‐ and epicardial border of the left ventricular wall distinguishable throughout the full cardiac cycle. Moreover, a frame rate of greater than 200 frames per second is essential [3, 4, 7] To prevent angular variation in the PSLAX view, which would have a significant impact on the LAA measurements, it is recommended that the data collection is conducted by the same observer. It is also crucial to take into account the specific animal model that is being utilized. As the flow profile across the MV is modeled by the change of volume in the left ventricle during diastole, it is not possible to adequately analyze models with valvular heart disease. In particular, it is not possible to detect stenoses or insufficiencies of the left ventricular valves. Moreover, we conducted this study using disease models characterized by rather homogeneous distortion of myocardial function in all segments of the left ventricle. Measurements in models exhibiting regionally inhomogeneous wall movement, such as myocardial infarction or postinfarction remodeling, may be significantly over‐ or underestimated due to the ventricular presentation in a single plane [4].

## 4 Materials and Methods

### 4.1 Concept of the NAEM Approach

Attempts to develop alternative approaches for measuring diastolic function parameters in mice can already be seen in the field of murine cardiac imaging using high‐temporal‐resolution cinematic magnetic resonance imaging (CINE MRI) [18, 19, 20]. Although CINE MRI offers high spatial resolution and tissue contrast, it lacks an application like that of PW Doppler in ultrasound for quantifying flow velocities as a function of time. Consequently, the aforementioned parameters cannot be assessed by measuring flow over the MV using CINE MRI. But by frame‐wise assessment of the left ventricular volume (LVV), the construction of both a *LVV*(*t*)‐curve and, through mathematical differentiation, a *dLVV*(*t*)/*dt* curve is possible. Here, the *dLVV*/*dt*(*t*)‐curve obtained by CINE MRI is the equivalent of the flow velocity profile (v(t)) over the MV as acquired with PW Doppler, allowing for the measurement of IVRT and *E*/*A* ratio. This approach rests on the assumption that the MV orifice area remains constant throughout the ventricular filling phase. This is due to the laws of volumetric flow rate:

(1) $$v\left(t\right)A=Q$$

(2) $$Q=\text{dLVV}\left(t\right)/\mathrm{dt}$$

and, therefore,

(3) $$v\left(t\right)A=\text{dLVV}\left(t\right)/\mathrm{dt}$$

In the above equations, *v* denotes flow velocity over the MV, *A* the cross‐sectional area of the MV, and *Q* the volume of blood entering the left ventricle per time. *v*(*t*) denotes the MV flow profile as a function of time acquired by PW Doppler, and *dLVV*(*t*)/*dt* is the change in LVV as a function of time. Given that *A* is assumed constant, it is anticipated that the parameter *dLVV*(*t*)/*dt* will change equally to MV flow velocity. Therefore, the usage of brightness‐mode (B‐mode) cine loops of the PSLAX provides an alternative to the established gold standard using PW Doppler in A4C view.

### 4.2 Animals

C57BL/6 mice at an age between 8 and 13 weeks were used for the investigation of diastolic function assessment. All described animal experiments were performed according to the European Animal Welfare Declaration and permission was granted by local authorities (Landesdirektion Sachsen, TVV49/2020, TVV16/2021).

### 4.3 Experimental Models

#### 4.3.1 Unimpaired Diastolic Heart Function

Male (*n* = 6) and female (*n* = 2) C57BL/6J mice were examined at an age of 8 weeks. These mice displayed an unimpaired heart function under baseline conditions [4, 10]. (Table 1—Unimpaired heart function).

#### 4.3.2 Chronic, Moderate Diastolic Dysfunction in HFpEF

A “two‐hit” mouse model of chronic HFpEF and moderate diastolic dysfunction was established in 8‐week‐old male C57BL/6N mice (*n* = 8), exposed to a high fat diet and L‐NG nitroarginine methyl ester (L‐NAME) added to the drinking water (0.5 g L^−1^) as published by Schiattarella et al. [21]. This model simulates a chronic condition of systemic vascular and metabolic stress mimicking key features of human HFpEF, including impaired myocardial relaxation due to increased myocardial stiffness. Male littermates (*n* = 6) with normal diet and without L‐NAME served as controls. After 5 weeks of treatment (age 13 weeks), animals underwent echo assessments. (Table 1—HFpEF).

#### 4.3.3 Acute, Severe Diastolic Dysfunction in HFrEF

Male (*n* = 6 per group) and female (*n* = 2 per group) C57BL/6J mice at an age of 8 weeks were injected with 5 μg lipopolysaccharide (LPS)/g body weight into the peritoneal cavity to induce a systemic inflammation response resulting in acute HFrEF and severe diastolic dysfunction [22, 23]. This model simulates the rapid and pronounced functional impairment seen in inflammatory or septic heart failure, with hallmarks of ventricular dilation, impaired contractility, and relaxation. Sex‐matched littermates injected with phosphate buffered saline (PBS) served as controls. The animals were examined 6 h after injection, the time point of expected maximal functional impairment. (Table 1—HFrEF).

### 4.4 Echocardiography

The echocardiographic studies were conducted by experienced, blinded operators using the VEVO 3100 imaging system (VisualSonics, Fujifilm) with a linear MX400 (30 MHz) ultrasound transducer and according to the recommendations of the European Society of Cardiology [4]. Animals were initially anesthetized with a mixture of 1 L/min O_2_ and 3%–4% isoflurane. After a sufficient level of anesthesia was reached, isoflurane was reduced to 1%–2% and animals were placed in a supine position on a heated examination platform. The chest fur was removed (Hair Removal Cream, Veet). Heart rate, respiratory rate, and body temperature were monitored using four limb electrodes as well as a rectal temperature probe. The parameters were kept within a physiological range by adjusting the isoflurane dosage and heating. When vital parameters were in a steady state the echo examination was started.

B‐mode cine loops in PSLAX as well as PW Doppler and Tissue Doppler cine loops in the A4C were recorded. In PSLAX attention was paid to a clear visibility of the aortic valve and the apex cordis throughout the cardiac cycle (Figure 1c). For MV flow assessment, the PW Doppler sample volume was placed at the tips of the MV in the A4C (Figure 1a), allowing for the measurement of transmitral blood flow velocities during diastole, capturing the early (*E*) and late (*A*) filling waves. Between both recordings, hemodynamic conditions were stable. Additionally, Motion‐Mode (M‐Mode) cine loops from the parasternal short axis (PSSAX) were recorded for phenotyping analysis. Experiments in the groups with unimpaired cardiac function and HFrEF were conducted by one investigator, whereas the HFpEF experiments were performed by another investigator.

Recorded cine loops were analyzed using the VEVO Lab software (VisualSonics, Version 2.1.0). Analyses of IVRT and *E*/*A* ratio for both measurement approaches were done by three observers, blinded to the experimental groups as well as to the results of the complementary method for diastolic function assessment. Noteworthy, two observers were instructed for the NAEM approach solely by reading a draft of the following methodology described in Section 4.7. Measurement of *E*–*A* peak interval and echocardiographic phenotyping analysis were conducted by one experienced observer, blinded to the experimental groups.

### 4.5 Measurement of *E*–*A* Peak Interval in PW Doppler Cine Loops

Measurements in PW Doppler cine loops (in A4C) were carried out by adding positive and negative “peak ± trace” to the recorded MV flow profiles. This tool in Vevo LAB traces the flow profile, helping the observer to identify the (positive) maximum flow velocities of the *E* and *A* waves (Figure 1b). The temporal interval between these peaks was quantified in three consecutive cardiac cycles at a temporal resolution of 2000 Hz for each recording. It was noted whether *E* and *A* wave were partially or totally fused. The corresponding heart rate was noted precisely from the respective segment of interest.

### 4.6 Assessment of IVRT and *E*/*A* Ratio in PW Doppler Cine Loops (Gold Standard)

As described in Section 4.5 “peak ± trace” was utilized in analyzing MV flow profiles. IVRT was identified as the time section between signals of aortic outflow (negative) and MV inflow (positive). *E* and *A* wave peaks were identified as the maximum (positive) flow velocity of early and late filling (Figure 1b). IVRT and *E*/*A* ratio were measured in three cardiac cycles at the time of maximum expiration.

### 4.7 Assessment of IVRT and *E*/*A* Ratio in B‐Mode Cine Loops (NAEM)

To process the obtained B‐mode cine loops in PSLAX, the Vevo Strain Analysis software, an additional analysis tool for VEVO Lab using speckle tracking, was applied as follows. An axis from the anterior to the posterior wall of the left ventricle (LV) was set manually to generate an anatomical‐M‐mode. Three cardiac cycles were selected and the endo‐ and epicardial borders were traced. By this, the software selected 25 evenly arranged regions of interest (ROIs) along the endocardial border (Figure 1c) and generated a continuous tracing of the left ventricular endocardium and, thus, the two‐dimensional LV area throughout the selected time interval. In case of incorrect tracing, throughout the selected cardiac cycles, the original ROIs were adjusted, judged by repeated visual inspection. Using the generated LV area values, LV volumes, LVV(t) curve, and *dLVV*(*t*)/*dt* curve were calculated by the software and displayed in a graph (Figure 1d). IVRT was identified in the *dLVV*(*t*)/*dt* curves between aortic outflow (indicated as sections of negative volume change) and MV inflow (indicated as sections of positive volume change). In this approach, the interval of interest was not necessarily depicted as a level section of no volume change. Rather, it was represented as a more shallow or irregular shaped section between sections with steep slopes of LVV change. However, the change in slope at the start and end of the isovolumic phase of LVV was typically so steep that the inclination could be reliably identified through visual inspection by the different observers (for untypical IVRT morphology, see Figure S2). Measuring the *E*/*A* ratio in the *dLVV*(*t*)/*dt* curves was achieved by identifying the points of maximal volume change during MV inflow of passive (first wave, *E*) and active (second wave, *A*) filling. The Vevo Strain Analysis allows measuring amplitudes of these curves (as in the *E*/*A* ratio measurement) by moving the mouse cursor along the appropriate point on the curve. The time and amplitude values are displayed. Time intervals (as in the IVRT measurement) can be quantified using two movable markers (at the beginning of the measurement, these can be found in red at the left and right edges of the graph).

### 4.8 Statistical Analysis

Statistical analyses were applied using GraphPad Prism 10.5.0. The two measurement approaches of the diastolic function parameters IVRT and *E*/*A* ratio were compared using a correlation analysis (Pearson's correlation) and a Bland–Altman analysis, a method specifically designed for evaluating the agreement between two measurement methods. For Bland–Altman analysis the difference between two measurements is plotted against the average of both measurements in absolute numbers and expressed in %. The average of these differences or “bias” and its deviation were used for evaluation. All analyses were done for the data of the three experimental groups separately as well as for combined data sets. In addition, the individual CV was calculated for both inter‐observer variability and the variability between the two approaches. For quantifying the relation of *E* and *A* wave fusion to heart rate a correlation analysis (Pearson's correlation) was performed for the *E*–*A* peak interval in comparison to the heart rate. By using a fitting regression, a heart rate threshold between well separated *E* and *A* waves and partial *E* and *A* fusion could be identified. Differences in regression slopes and elevation were assessed by an ANCOVA, testing the interaction between group and covariate (*F*‐test).

All data sets were tested for normal distribution using the D'Agostino and Pearson's test. *p*‐Values < 0.05 were deemed significant.

## 5 Conclusion

Transmitral Doppler–based assessment of diastolic function in murine echocardiography is subject to major limitations. Here we quantitatively characterized heart rate‐dependent *E* and *A* wave fusion and identified a threshold of approximately 460 bpm in mice with unimpaired cardiac function and HFpEF, whereas advanced HFrEF exhibited disease‐driven alterations in filling dynamics without clear relation to heart rate. These findings further restrict the interpretability of *E*/*A*‐derived parameters in experimental settings.

To address this limitation, we introduce the speckle tracking–based NAEM approach, which enables reliable IVRT measurement from B‐mode PSLAX cine loops and shows strong agreement with the Doppler‐derived reference method. By allowing IVRT quantification independent from mitral flow profile recordings, NAEM‐IVRT could be integrated into established algorithms for diastolic function assessment, facilitating comprehensive evaluation using a single imaging modality (B‐mode PSLAX) and supporting retrospective analysis of existing datasets.

Together, these findings contribute to a standardized, quantitative, and technically accessible framework for diastolic function phenotyping in experimental cardiovascular research using a speckle tracking–based NAEM approach.

## Author Contributions

**Michael Marterstock:** conceptualization, methodology, validation, formal analysis, investigation, writing – original draft, writing – review and editing, project administration. **Antje Schauer:** validation, investigation, writing – review and editing. **Alina Maslakova:** validation, investigation, writing – review and editing. **Annett Opitz:** investigation. **Peter Dieterich:** methodology, writing – review and editing. **Stephan Speier:** resources. **Susanne Kämmerer:** resources, writing – review and editing. **Peter Mirtschink:** methodology, resources, writing – review and editing. **Irakli Kopaliani:** resources, writing – review and editing, supervision, funding acquisition. **Andreas Deussen:** conceptualization, methodology, resources, writing – review and editing, funding acquisition, supervision.

## Funding

This work is supported by research grants of the German Research Foundation (DFG grant DE360/10‐1), Deutsche Gesellschaft für Kardiologie‐Herz und Kreislaufforschung e.V. (DGK—“Otto‐Hess” thesis scholarship to Michael Marterstock), Deutsche Herzstiftung e.V. (to Susanne Kämmerer) and the Faculty of Medicine Carl Gustav Carus, Dresden University of Technology as well as Stiftung zur Förderung der Hochschulmedizin Dresden (“Keep‐on‐track”‐Funding to Michael Marterstock). These fundings had no impact on study design; collection, analysis, and interpretation of data; manuscript writing; and decision to submit the article for publication.

## Ethics Statement

All described animal experiments were performed according to European Animal Welfare Declaration and permission was granted by local authorities (Landesdirektion Sachsen, TVV49/2020, TVV16/2021).

## Consent

The authors have nothing to report.

## Conflicts of Interest

The authors declare no conflicts of interest.

## Supporting information


**Figure S1:** Comparison of gold standard approach versus NAEM approach for IVRT measurement.

**Figure S2:** Two examples of untypical IVRT morphology in the NAEM‐IVRT approach for (a) unimpaired heart function and (b) HFrEF.

**Table S1:** Results of Pearson's correlation and Bland–Altman analysis for IVRT measurement comparison.

## Data Availability

The data that support the findings of this study are available from the corresponding author upon reasonable request.
