# Supplementary figures and images for: Addressing Key Limitations of Diastolic Function Assessment in Mouse Echocardiography by Enabling Robust Retrospective Analysis From a Standard Imaging View
Source: Acta Physiol (Oxf). 2026 May 14;242:e70250. doi: 10.1111/apha.70250 (PMC13176516; doi:10.1111/apha.70250)

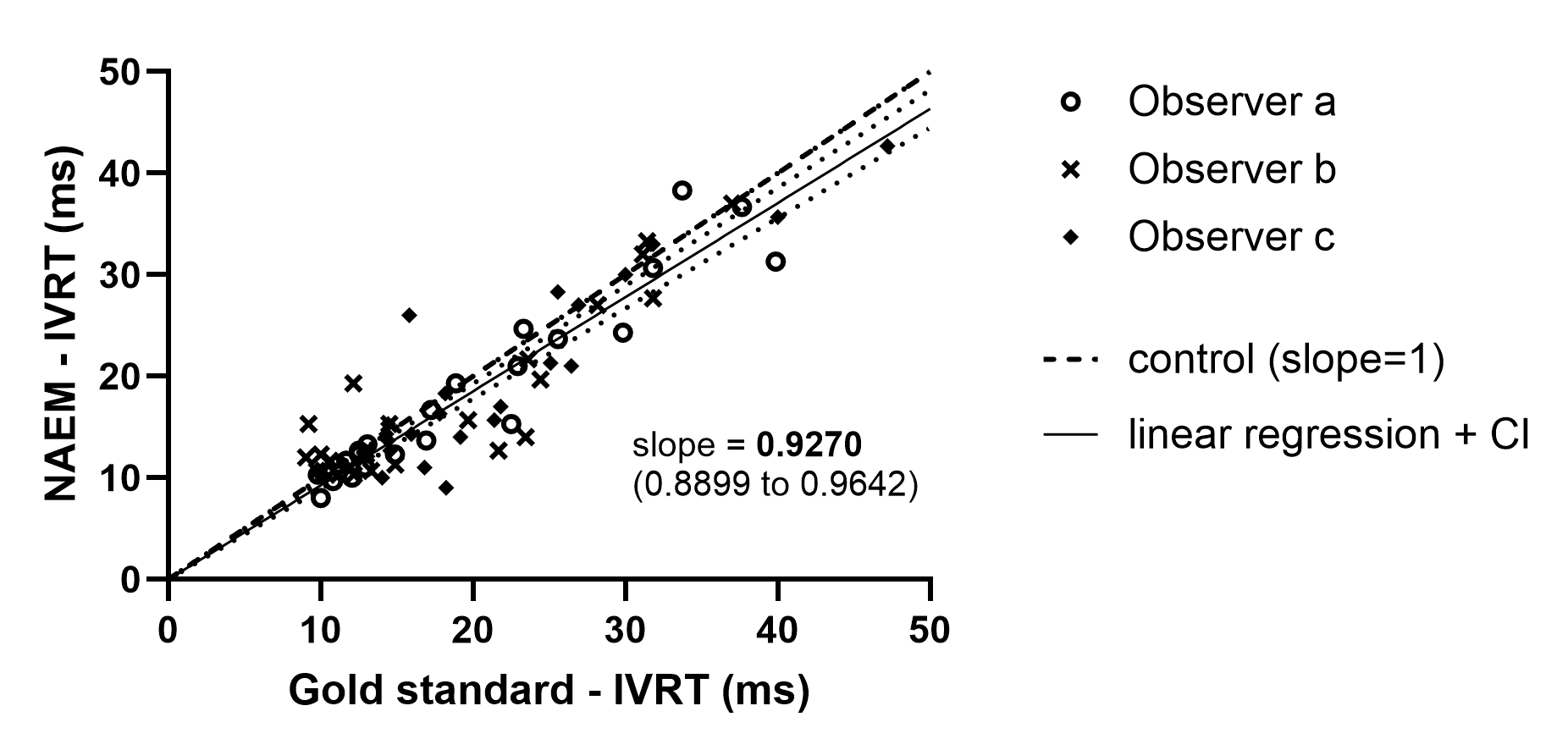

Supplement: Supplementary file 1 — Figure S1: Comparison of gold standard approach versus NAEM approach for IVRT measurement. [file APHA-242-e70250-s002.tif]

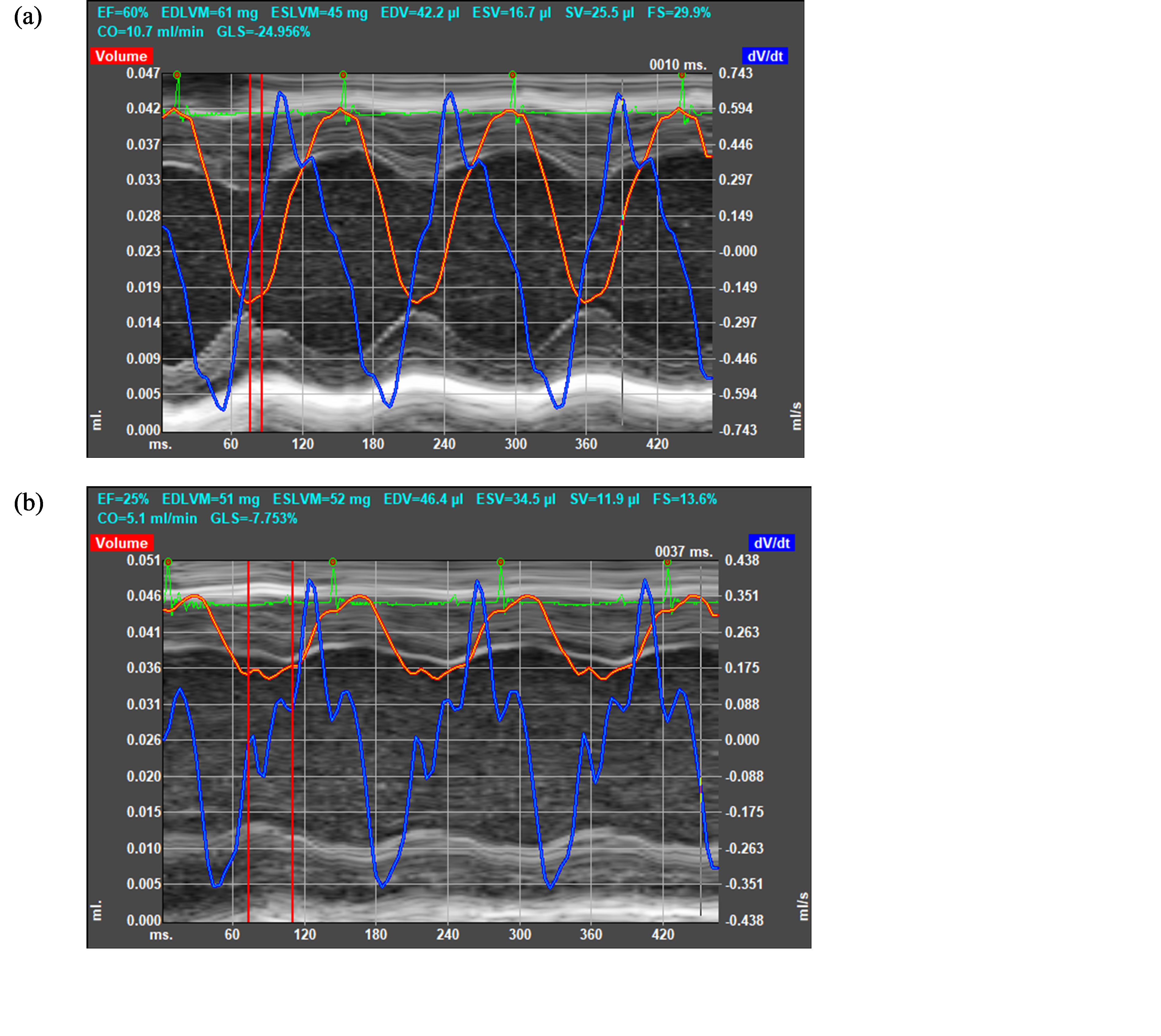

Supplement: Supplementary file 2 — Figure S2: Two examples of untypical IVRT morphology in the NAEM‐IVRT approach for (a) unimpaired heart function and (b) HFrEF. [file APHA-242-e70250-s003.png]
