# Supplementary material for: Addressing Key Limitations of Diastolic Function Assessment in Mouse Echocardiography by Enabling Robust Retrospective Analysis From a Standard Imaging View
Source: Acta Physiol (Oxf). 2026 May 14;242:e70250. doi: 10.1111/apha.70250 (PMC13176516; doi:10.1111/apha.70250)
Supplement: Supplementary file 3 — Table S1: Results of Pearson's correlation and Bland–Altman analysis for IVRT measurement comparison. [file APHA-242-e70250-s001.docx]

**Table S1** Results of Pearson´s correlation and Bland-Altman analysis for IVRT measurement comparison

| **Pearson´s correlation** | Observer a  (n=24) | Observer b  (n=24) | Observer c  (n=23) | Combined  (n=71) |
| --- | --- | --- | --- | --- |
| p-value | <0.0001 | <0.0001 | <0.0001 | <0.0001 |
| r² | 0.9139 | 0.8006 | 0.8188 | 0.8485 |
| r Confidence interval (95%) | 0.8994 to 0.9810 | 0.7689 to 0.9539 | 0.7858 to 0.9593 | 0.8761 to 0.9502 |
| **Bland-Altman analysis: NAEM approach vs. average** |  |  |  |  |
| Bias (%) | -7.8 | -3.1 | -11.1 | -7.3 |
| SD of bias (%) | 12.2 | 24.7 | 22.5 | 20.5 |
| Bias (ms) | -1.39 | -0.83 | -1.83 | -1.34 |
| SD of bias (ms) | 2.74 | 3.90 | 3.98 | 3.55 |

(individual data points from each observer for 24 animals from 3 experimental groups)
